# Real or bogus: Predicting susceptibility to phishing with economic experiments

**DOI:** 10.1371/journal.pone.0198213

**Published:** 2018-06-27

**Authors:** Yan Chen, Iman YeckehZaare, Ark Fangzhou Zhang

**Affiliations:** School of Information, University of Michigan, Ann Arbor, Michigan, United States of America; University of Texas at San Antonio, UNITED STATES

## Abstract

We present a lab-in-the-field experiment to demonstrate how individual behavior in the lab predicts their ability to identify phishing attempts. Using the business and finance staff members from a large public university in the U.S., we find that participants who are intolerant of risk, more curious, and less trusting commit significantly more errors when evaluating interfaces. We also replicate prior results on demographic correlates of phishing vulnerability, including age, gender, and education level. Our results suggest that behavioral characteristics such as intolerance of risk, curiosity, and trust can be used to predict individual ability to identify phishing interfaces.

## Introduction

The rapid evolution of information technology in recent decades has vastly changed our daily lives, from how we communicate with others to how we shop. However, the benefits of these technologies have come at the expense of our privacy and security. According to a recent survey by Wombat Security Technologies, the financial loss incurred due to successful phishing amounts to $4 million for an average 10,000-employee company. (see https://www.wombatsecurity.com/about/news/report-phishing-costs-average-organization-37-million-year. Retrieved on September 17, 2016.)

Various resources have been dedicated to reduce phishing susceptibility and most of these efforts focus on enhancing user awareness through warning systems. However, preventing a phishing attack depends not only on the technological sophistication of a system, but also on the personal characteristics that make some users more vulnerable to phishing attempts. Any phishing intervention requires an understanding of the personal characteristics that relate to the ability to recognize phishing attempts [1]. Indeed, when J.P. Morgan Chase & Co. sent a fake phishing email to its 250,000 employees, it found that 20% clicked on the bogus link. (see https://www.wsj.com/articles/banks-battle-staffers-vulnerability-to-hacks-1450625921. Retrieved on September 17, 2016.)

This paper presents a study that predicts participants’ ability to distinguish phishing attempts from legitimate interfaces using behavioral attributes elicited in a series of economic experiments. Our participants are Business and Finance (B&F) staff members at a large public university in the U.S., whose work is commonly involved with sensitive data in the organization. The staff members are encouraged to participate in a phishing awareness education module developed by the Information and Infrastructure Assurance (IIA) program at the university. We add two research components to the education module. Before the education module, each participant takes a security quiz which includes seven questions, each of which asks the subjects to determine the legitimacy of an interface (website or email). While the subjects are not explicitly asked to click a link deemed as legitimate, their answers are used to determine their ability to identify phishing attempts. After completing the training module, they are invited to participate in a series of economic games designed to measure their risk attitude, trust and curiosity.

Our results show that, on average, our subjects correctly answer five out of seven questions on the security quiz. However, we also find that nearly half of our subjects erroneously identify at least one legitimate interface as phishing attempt, and more than 30% misidentify both legitimate and bogus interfaces. We also find that those participants who are intolerant to risk score significantly worse on the security quiz due to their misidentification of legitimate interfaces as phishing attempts. Furthermore, we find that less trusting participants are more likely to be uncertain about a legitimate interface while more curious participants tend to mistake a legitimate interface as a phishing attempt. Finally, we confirm previous finding that age and gender are related to phishing susceptibility.

To our knowledge, this is the first study to provide predictions on individual susceptibility to phishing with their measured behavioral traits in a controlled laboratory experiment. Compared with prior studies on phishing—most of which use hypothetical scenarios, survey questions, or fake attacks to determine participants’ behavioral attributes [2, 3], our experiments can generate more precise and stable measurements of individual preferences (see [4–6] for surveys of the three games) and provide reliable out-of-sample predictions of individual behavior in the field [7, 8].

Second, we contribute to the literature by distinguishing between two types of negative consequences from succumbing to phishing attempts. First, we examine the effect of regarding a phishing email or website as legitimate (a *false negative*). In addition, we examine the effect of regarding a legitimate email or website as a phishing attempt (a *false positive*). The latter has been largely ignored in the literature, with the exception of [2] and [9], who find that anti-phishing education reduces the participants’ tendency to click on legitimate links. To our knowledge, ours is the first attempt to use behavioral traits to predict the tendency of an individual to regard a legitimate website as a phishing attempt using behavioral attributes.

In the recent years, attackers sometimes simulate the university authorities’ email templates in an attempt to convince staff members to click on the phishing links in their emails. Since B&F staff have access to highly sensitive information, the authorities encourage them not to click any link until they verify its legitimacy. As a result, the staff become more cautious and sometimes even ignore legitimate emails, which makes false positive errors especially harmful as tasks delegated are sometimes delayed or ignored. In a broader context, many charitable organizations find that their email communications to potential donors or contributors are often ignored, although in some cases these emails might have been conveniently ignored. To this end, in the examples provided in the security quiz, we include both emails appearing to be generated by university authorities and those simulated with phishing links. This makes it possible to measure the participants’ tendencies toward trusting phishing emails and ignoring legitimate ones.

Since our study is conducted together with a security education program, participants might be more cautious than they normally are. That is, they might be more suspicious about the legitimacy of an interface than under the regular work environment. This has two implications regarding the external validity of our study. First, the predictive power of inconsistency, intolerance to risk, trust and curiosity on the tendency to misidentify a legitimate interface as bogus might be weaker in regular context. Second, given that participants might normally be less cautious, the attributes that appear to predict the tendency to fall for phishing, such as gender and age, would be more pronounced in regular work environment.

## Experimental design

To conduct our study, we partner with the Office of Information Security Assurance (IIA) at a large public university in their development of a phishing education module for the B&F staff. Before launching the study, the Chief Financial Officer of the university sends an email announcement to the B&F supervisors and staff, respectively, encouraging them to participate in the education module and research study. On April 14, 2016, we launched the study with an email to all B&F staff members that includes a link to the study website. The staff members can access the site with their university account, using the university authentication system. The study lasts for one month, during which a staff member can log onto our website at any time to participate. Out of the 3,190 B&F staff members, 1,201 participate in both the security quiz designed by the researchers and the security education module designed by the IIA staff. Of these, 811 staff members also participate in our economic experiment. Participants spend on average 10 minutes and 2 seconds (*s*.*d*. = 6.15 minutes) finishing all the components, with average earnings of $17.04 (*s*.*d*. = $6.04) from the economic games.

Our study consists of two parts: an information security quiz with seven phishing-related questions and an incentivized economic experiment. To measure participants’ risk attitude as well as their trust and curiosity, we include three economic games in our experiment: a lottery choice game [10], a gamble game [11], and a trust game [12]. Each participant who chooses to participate in the experiment plays all three games and is paid at the end of the experiment based on her decisions as well as those of her match. The order of the games is randomized such that each participant is randomly assigned one of six possible orders.

### The information security quiz

For the first part of our study, we present participants with seven questions, each with an interface (website or email), which is either legitimate or bogus. We ask our participants to judge the legitimacy of the interface in each of the seven questions. The questions in the quiz are selected from two sources: (1) recent successful phishing attacks (such as university library access renewal, notification of a shared document, or an alert message from a bank account) and (2) questions with high error rates from a fall 2015 information security quiz administered to 5,892 students at the university. The seven quiz questions are provided in the Supporting Information. According to the IIA office, in recent years, about 1,500 email accounts at the university is compromised due to successful phishing attacks. We draw quiz questions directly from this rich source of successful attacks.

To evaluate individual tendency to commit both false positive and false negative errors, we include five bogus and two legitimate interfaces in our quiz. The distribution of the two types of interfaces is unbalanced, as IIA was more concerned about staff members clicking on bogus links. One consequence of this unbalanced distribution is that we might observe more false negatives simply as a result of subjects making random choice. Furthermore, the order of the security questions is not randomized. Combining the first two questions into the “early group”, the three questions in the middle to the “middle group”, and the last two questions to the “late group”, we find that the percentage of correct answers between the three groups are significantly different from each other (*p*-value < 0.001, paired t-tests). Since the order is not randomized, we cannot differentiate between order effect or the inherent difficulty level of the question.

After completing the security quiz, participants receive their quiz score and go through an interactive phishing awareness education module, which provides them with instructions on how to identify phishing attempts. This education module is designed by the IIA, and is not part of our study design. At the end of the education module, participants are invited to continue and complete an incentivized economic experiment with three games.

### Economic games: Risk, trust and curiosity

Our game selection is based on the large literature on decision making under risk and uncertainty. In a simple expected utility model where the state of the world is uncertain, i.e., whether an interface is real or bogus, a decision-maker (DM) optimizes by choosing whether to trust the interface or not. We use *s* ∈ {*b*, *r*} to represent the state of the world, bogus or real, and *p*(*b*) to represent the subjective probability that the interface is bonus. A DM’s state dependent utility function is represented by *u*(*a*|*s*), where *a* ∈ {0, 1} represents the DM’s binary action, to click or not to click. In our experiment, the action is whether to evaluate the interface as real or bogus. Therefore, a DM will maximize the following standard expected utility function:

$$\begin{array}{c} EU(a,s)=p(b)u(a|b)+(1-p(b))u(a|r). \end{array}$$ (1)

The optimal action will depend on several factors. The first factor is the curvature of the utility function, *u*(⋅), i.e., a person’s risk preference. Therefore, we need to measure the DM’s risk preferences. The second factor is the DM’s prior belief about the likelihood that the interface is bogus, which implies that we need to measure a person’s trust. The third factor is the DM’s curiosity, which is a desire to close the information gap, which is not in the standard model, but important to measure given the context.

### Eliciting risk preferences

To elicit our participants’ risk preferences, we use a lottery choice game modified from [10]. In this game, subjects participate in ten lotteries, each containing an A and B option. Both options have a high and low payment, and each is realized with a certain probability. The probability of receiving the high payment increases from 10% in the first lottery to 100% by the last. The ten lotteries vary only in their probability of receiving the payoffs. S1 Fig displays the interface for the lottery choice game. Given this structure, a risk-averse expected utility maximizer would start by selecting option A in the first lottery and switch to option B at some point. We measure risk attitude as the switch-over point and compute the implied risk parameter from this switch point. For each of the ten decisions, participants indicate which lottery (the A or B option) they want to play by clicking either option A or option B.

To check the reliability and consistency of our measure for risk attitude, we also have subjects take part in the gamble game by [11]. Compared to the lottery choice game, the gamble game provides a coarser but more intuitive measure of risk preferences. In this game, participants are presented with a table containing nine 50-50 lotteries based on the outcome of a coin toss. The first lottery corresponds to a sure amount of $4 regardless of whether the outcome is heads or tails. In subsequent lotteries, the high payoff increases by $1 and the low payoff decreases by $0.5. The participants are asked to choose one of the nine lotteries and their payoff will be determined by the toss of a fair coin. S1 Fig provides the interface for the gamble game.

Based on our standard expected utility model, we formulate the following hypothesis on the effect of risk aversion on errors in the security quiz.

**Hypothesis 1** (Risk). More risk averse individuals are more likely to make false positive errors.

### Eliciting level of curiosity

At the end of the lottery choice game, the computer randomly selects one lottery to determine a participant’s earnings. Participants are told their final earnings but not which lottery is eventually chosen. Since the participants have already made their decisions and learned how much they have earned, the information on which lottery has been chosen has no instrumental value and the participants’ willingness to pay (WTP) for it provides a measure for curiosity [13–15]. To elicit the participants’ WTP for this non-instrumental information, we use the Becker-DeGroot-Marschak procedure [16, 17] described as follows. The participants choose a number between 0 and 4 to indicate their WTP to learn the lottery that was selected. The computer then randomly picks a number uniformly between 0 and 4. If the number picked by the computer is less than the participant’s WTP, the participant pays a price equal to the number and receives the lottery information. If the randomly-selected number is greater than the participant’s WTP, she pays nothing and receives no information. S1 Fig displays the interface for our curiosity measure. In sum, the lottery choice game provides a measure of both risk preference and curiosity.

Since our experiment does not require subjects to click on any link, we do not have a strong prior belief about the direction curiosity might influence their performance on the security quiz. Therefore, we formulate the following hypothesis.

**Hypothesis 2** (Curiosity). Individuals’ level of curiosity affects their performance on the security quiz.

### Eliciting trust

We measure the level of trust by the game developed by [12]. In a classic trust game, participants are randomly matched into groups of two where each player is given $5 and assigned the role of either an investor or a responder. In this game, an investor can transfer any amount from her initial endowment to the responder. The amount is then tripled by the experimenter before it is transferred to the responder. The responder can then transfer any amount back to the investor, including using her own endowment. S1 Fig in the Supporting Information display the interface for the trust game from the investor and responder’s perspectives, respectively. While the trust game is primarily used to measure trust and trustworthiness in the literature, more careful controls reveal that the amount invested reflects a combination of trust and altruism, whereas the amount returned reflects a combination of trustworthiness (reciprocity) and altruism [18]. In our version of the game, we use the strategy method where each participant submits her decisions as both an investor and a responder who decides how much to return contingent upon each possible investment.

To determine payoffs, we randomly match a participant to one of 41 subjects from a pilot session of the study and randomly assign the roles of the investor and the responder. Earnings are then calculated according to each participant’s decision for the role to which she has been assigned.

In our standard expected utility model, trust is assumed to affect a decision-maker’s prior belief that an interface is bogus. Based on the model, we formulate the following hypothesis on the effect of trust on errors in the security quiz.

**Hypothesis 3** (Trust). More trusting individuals are (a) less likely to make false positive errors, but (b) more likely to make false negative errors.

## Data

A total of 1,201 B&F staff members participate in the information security quiz, among whom 811 complete the economic experiment described above. In addition to the behavioral data, we obtain the participants’ demographic data from the university’s HR department. Among the 1,201 staff members who take the quiz, we are able to link 1,125 participants’ behavioral data with their demographic information, including age, gender, ethnicity, education background and annual salary. Because the demographic dataset is obtained two months after the end of the study, it does not contain information for the 76 members due to staff turnover during those two months.

Table 1 presents the summary statistics of demographic characteristics for the participants who take only the quiz, the participants who take both the quiz and the game, and the non-participants of the study. We find that those who participate in our study represent a more balanced gender composition, larger proportion of whites, higher education levels, and higher annual salary than those who do not. Even though we do not have occupation information, it is plausible that those with higher salaries and better education have higher positions in the organization, and therefore might be more wary of being phished, which might translate into more false positive errors. This biased selection into the quiz implies that we might observe more false positives in our sample than in the general population, which limits the external validity of the study.

**Table 1:** Demographic characteristics of participants.

	Participants: (Opted In)			Nonparticipants: (Opted Out)
	Quiz only	Quiz & Games	Pooled	
Age	50.25***	46.24	47.53	46.68*
	(10.31)	(11.02)	(10.95)	(11.40)
Female (%)	53.74	55.24	54.76	36.24***
White (%)	83.93	84.03	84.00	75.98***
High School or Lower(%)	13.85**	10.99	11.91	32.64***
Bachelor (%)	64.54	64.53	64.53	57.22***
Post Graduate (%)	21.64	24.48	23.56	10.14***
Salary (thousands)	76.27	73.74	74.55	61.01***
	(37.38)	(38.72)	(38.29)	(30.84)
# obs	361	764	1125	2298

Notes: ***, ** and * denote significance at the 1%, 5% level and 10% level using the Wilcoxon rank-sum tests (for Age and Salary) and the binomial tests (for Female, White, High School or Lower, Bachelor, and Post Graduate) testing the equality of demographic characteristics 1) between participants who take only the quiz and participants who take both the quiz and the game, and 2) between participants and non-participants.

Our main dependent variable is the participant quiz score, which is the number of correctly answered questions. The average score of the 1,201 B&F staff members is 4.97 out of 7.00, with a standard deviation of 1.25. More than 95% of the participants answer at least three questions correctly and about 11% of them answer all the seven questions correctly. We first investigate whether there is any sample selection bias by comparing the quiz scores of those who participate in the economic experiment and those who do not.

**Fig 1:**
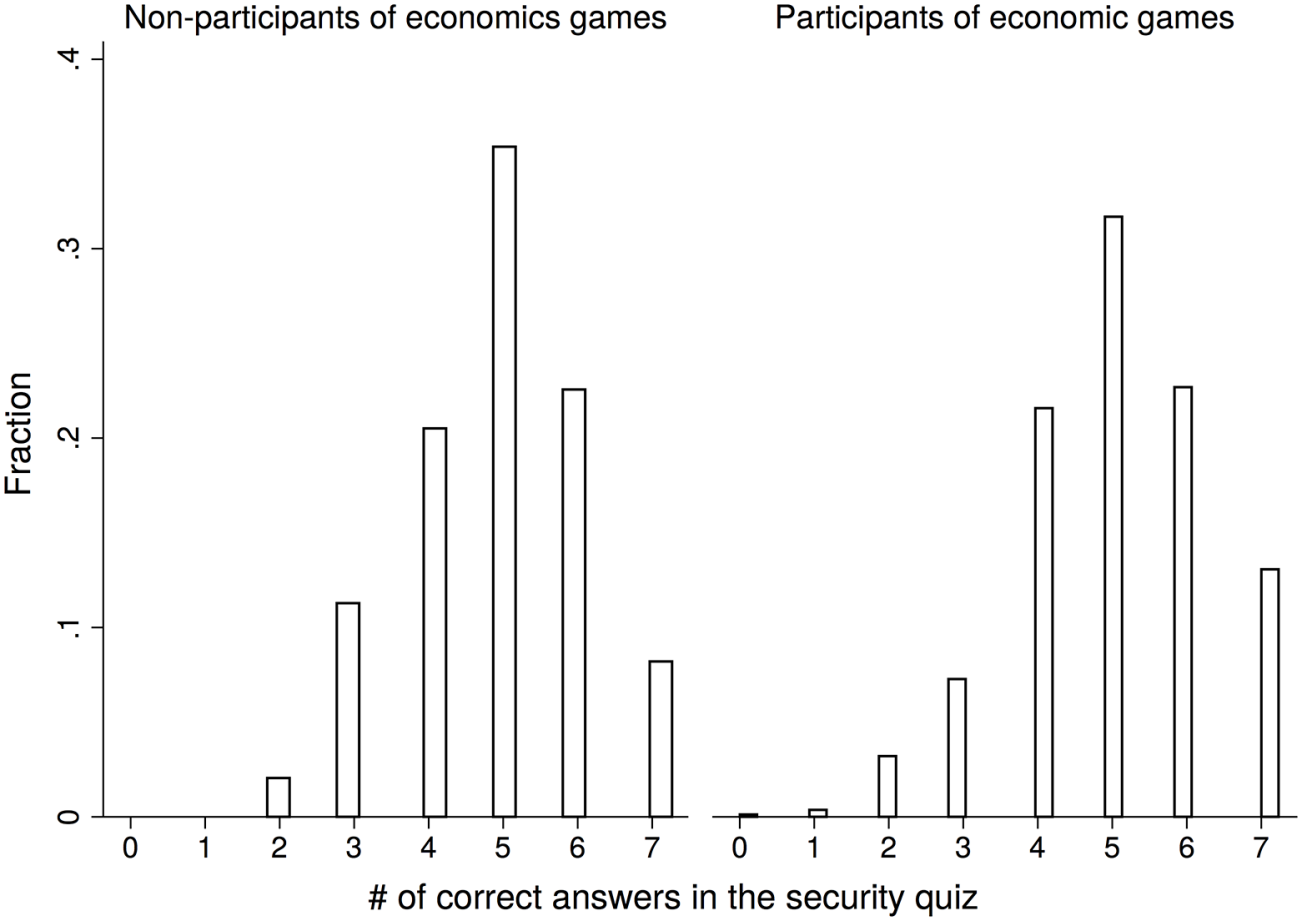
Quiz score distributions among non-participants (left panel) and participants of economic games (right panel).

From Fig 1, we see that the average number of correctly answered questions is 4.90 for those who do not participate in the economic games compared to 5.01 for those who do. A permutation test of the two samples indicates that the null hypothesis that the quiz scores for the non-participants and the participants come from the same population cannot be rejected (*p* = 0.10, two-sided). Furthermore, a Kolmogorov-Smirnov test reveals no significant difference in the score distributions for the two groups (*p* = 0.53, two-sided). The null hypothesis of the normality of the score distribution is rejected for both samples at 1% significance by the Kolmogorov-Smirnov tests.

We next examine the number of false positives (misidentifying a legitimate site) and false negatives (misidentifying a bogus site) in our quiz responses. Among the seven questions contained in the quiz, two reflect legitimate interfaces while five are bogus interfaces. Over 40% of our participants correctly identify all five phishing interfaces. Again, we find no significant difference in the number of false negatives between those who participate in the economic games and those who do not (*p* = 0.99, two-sided Kolmogorov-Smirnov test). In comparison, we find that nearly half our participants erroneously regard one legitimate interface as a phishing attempt and more than 30% misidentify both. Interestingly, those who do not participate in our economic games are marginally less likely to make false positive errors than those who do participate (*p* = 0.08, two-sided Kolmogorov-Smirnov test). One possible explanation is that the subjects who self-select into the economic games are more serious about the entire study and hence more cautious about the security quiz.

The data and analysis code reported in this paper have been deposited in the open Inter-university Consortium for Political and Social Research (ICPSR) data repository (http://doi.org/10.3886/E101360V1). The protocol for this study is available at dx.doi.org/10.17504/protocols.io.n74dhqw.

The source code of the web application developed for the purpose of this experiment is available at https://github.com/ImanYZ/PhishingExperiment.git.

Experimental instructions in the form of screen shots are provided in the Supporting Information section.

## Results

### Risk attitude

We measure participants’ risk preferences with two games. First, in the gamble game, we measure risk preference by holding the probabilities constant while varying the stake of the monetary reward. Our results for the gamble game show that more than 20% of our subjects choose the ninth, which gives the largest prize of $12 with a 50% chance. On the other hand, more than 31% of our subjects choose the first gamble, which offers a fixed payment of $4 with no risk.

In comparison, the lottery game measures risk preference by varying the probabilities associated with a series of lotteries while holding the stakes of each lottery constant. We measure risk preference as the point when a participant switches from option A to option B. Following [10], we call those subjects who switch only once *consistent* subjects. Our results show that 66.5% of our participants are consistent. Among our inconsistent participants, we find that 28.8% switch multiple times and 10.2% choose option A in the last row. This inconsistency may result from a lack of understanding or from indifference, in the case of multiple switching points. Indeed, inconsistency is commonly observed across various populations, typically correlated with the level of education. While it is typically low among college students (e.g., 5.8% among Danish undergraduates by [4]; 13% among U.S. undergraduates by [10]), it is higher among working adults, especially those with lower levels of education (e.g., 36% among Shanghai workers with a high school education by [19], 41% among fishermen in South Africa by [20], 55% among residents in Rwanda by [21]).

Table 2 displays the proportion of the subjects switching from option *A* to *B* at each lottery. Each row represents the interval estimate for the implied constant relative risk aversion (CRRA) coefficient and the risk preference classification according to [10]. In the last column, we report the proportion according to the choices from the gamble game. We find that nearly 10% of our subjects are risk loving, 11% are risk neutral, and 80% exhibit varying levels of risk aversion. Roughly 60% of the subjects switch to option B between the fifth and the seventh lottery, indicating a constant relative risk aversion coefficient in the range of *r* ∈ [0.1, 0.9], which is in line with measurements from prior literature [10].

**Table 2:** Risk preference calibration and classification using consistent subjects.

Lottery	CRRA Interval	Risk Preference Classification	Proportion from Lottery	Proportion from Gamble
1	*r* < −1.6	highly risk loving	7.9	n/a
2	−1.6 ≤ *r* < −1.1	very risk loving	0.9	n/a
3	−1.1 ≤ *r* < −0.5	risk loving	0.9	n/a
4	−0.5 ≤ *r* < 0.1	risk neutral	11.0	27.5
5	0.1 ≤ *r* < 0.3	slightly risk averse	20.0	0
6	0.3 ≤ *r* < 0.6	risk averse	21.3	18.0
7	0.6 ≤ *r* < 0.9	very risk averse	17.1	3.2
8	0.9 ≤ *r* < 1.3	highly risk averse	7.6	11.5
9	1.3 ≤ *r* < 1.8	extremely risk averse	1.3	0
10	1.8 ≤ *r* < 2.7	intolerant of risk	11.5	39.7

Notes: n/a indicates that risk preference under this category cannot be captured by the gamble game by construction.

Our results from the gamble game are consistent with those from our lottery game. Specifically, we find that the Spearman’s rank correlation coefficient between the switching point in the lottery and the choice number of the gamble is -0.28 (*p* < 0.01). Using a structural estimation, we find that the correlation between the CRRA coefficients inferred from the participants’ choices in the two games is 0.38 (*p* < 0.01).

**Fig 2:**
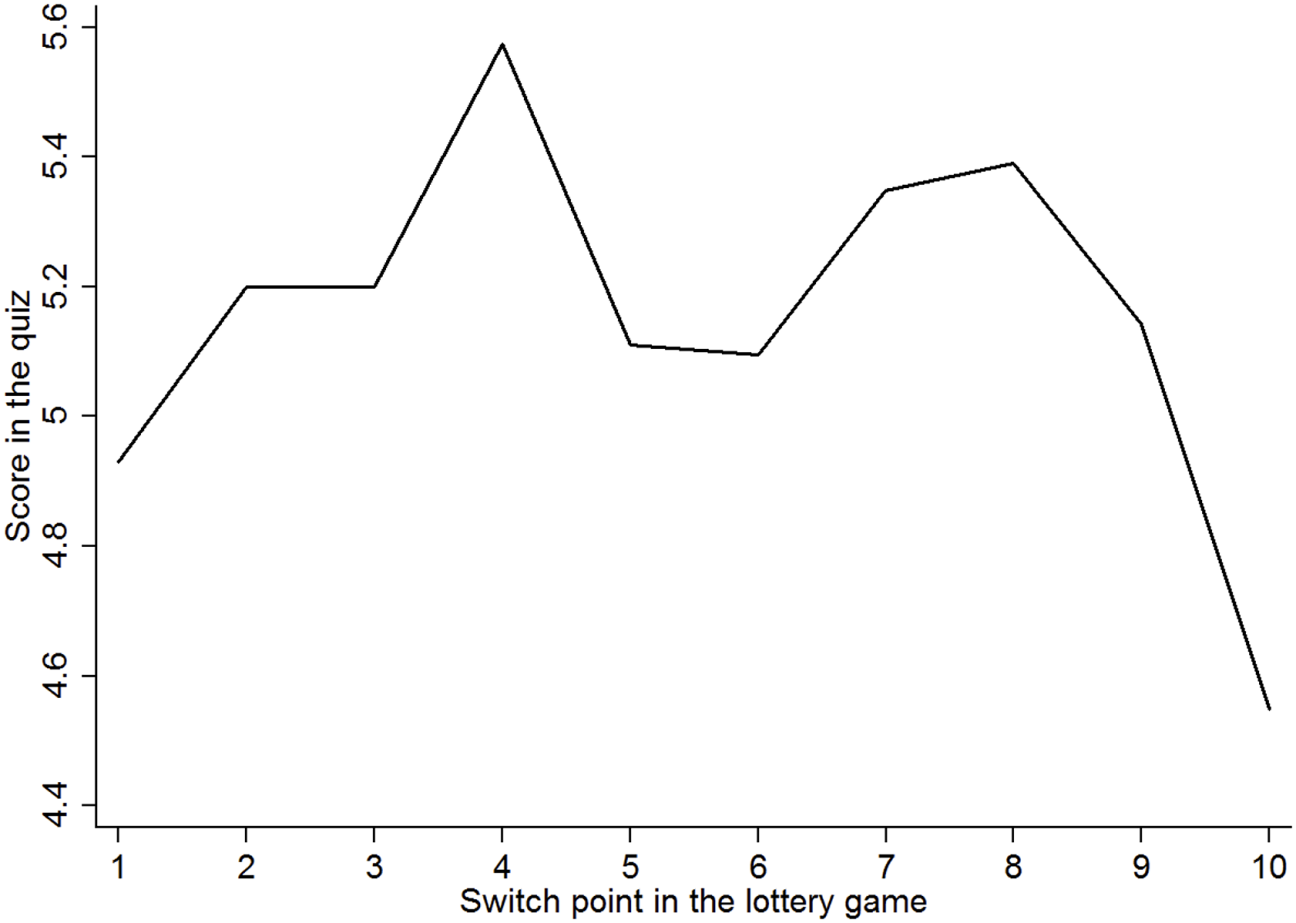
Average score in the quiz.

One interesting pattern observed in both games is that a substantial number of our subjects exhibit intolerance to risk by choosing the option that carries no uncertainty involved. In the lottery game, 11.5% of our subjects switch to option B in the last row, whereas no more than 1% of the subjects do so in the original lottery experiment by [10]. Similarly, about one third of the subjects choose the first lottery in the gamble game, compared to 10% of those in the study by [11]. A Spearman’s rank correlation test also indicates that choosing first gamble in the gamble game is significantly correlated with switching at the last row (*p* < 0.01). In Fig 2, we depict the average quiz score for the subgroup of consistent participants based on the switching point in the lottery game. This figure shows that those who are intolerant to risk are less likely to identify interfaces correctly.

To understand how lottery choice is associated with the performance in the quiz, we perform regression analysis using 1) average score in the quiz, 2) number of false positives and 3) number of false negatives as the outcome variables. For each regression, we perform the analysis for all participants as well as those whose choice is consistent in the lottery. We report the regression analysis for two specifications: OLS and ordered logit models. We choose OLS as its coefficients are easy to interpret, whereas ordered logit models have the advantage of not imposing linearity in magnitude. We report the estimates for the coefficients in the OLS and ordered logit models. The odds ratio for the latter is provided in S1 Table in the Supplemental Information. Our results are also robust when we use the corresponding Tobit models that account for truncation at corner cases.

**Result 1** (Risk). In the lottery game, inconsistent individuals perform significantly worse in the security quiz than consistent ones. Among the latter, individuals who are intolerant of risk achieve a lower score on the security quiz due to a greater number of false positive errors, whereas the variation in risk attitude does not predict performance on the security quiz.

**Support**. The average quiz score among consistent subjects is 5.142, which is significantly higher than that of the inconsistent subjects, 4.725 (p-value <1% using a t-test). Among subjects who switch to option B in one of the first nine lotteries, the average quiz score varies between 4.9 to 5.6 correct answers, compared to 4.5 correct answers for those who switch to option B in the tenth lottery. Column 3 and 4 in Table 3 report the OLS and ordered logit regressions over risk attitude measured by the switching point and a dummy variable which equals one if a subject switches to option B in the last row and zero otherwise. The parameter estimates suggest that switching to option B in the last lottery significantly decreases the score by nearly 0.60, with 0.38 of the decrease attributed to false negatives (see Table 4) and 0.22 to false positives (see Table 5). The variation in the level of risk aversion, however, has an economically and statistically insignificant impact on the quiz score.

**Table 3:** Regression of average score in quiz on behavioral attributes.

	All Participants		Consistent Participants	
	OLS	Ordered Logit	OLS	Ordered Logit
	(1)	(2)	(3)	(4)
consistency	0.224**	0.283**		
	(0.100)	(0.145)		
switching point			0.033	0.054
			(0.031)	(0.045)
**I**{switch at 10}			-0.599***	-0.790**
			(0.230)	(0.327)
curiosity	-0.030	-0.061	-0.071*	-0.120**
	(0.030)	(0.045)	(0.038)	(0.056)
trust	0.019	0.024	0.038	0.050
	(0.028)	(0.041)	(0.035)	(0.049)
age	-0.012***	-0.021***	-0.010**	-0.018**
	(0.004)	(0.006)	(0.005)	(0.007)
female	-0.520***	-0.805***	-0.533***	-0.880***
	(0.094)	(0.139)	(0.114)	(0.167)
*R*^2^	0.080		0.089	
Pseudo *R*^2^		0.027		0.033
# obs	764	764	506	506

Notes: In the ordered logit models (columns 2 and 4), the dependent variable is ordered according to the number of false positives, with zero being the lowest and two being the highest. *, ** and *** denote significance at the 10%, 5%, and 1% level, respectively.

**Table 4:** Regression of number of false positives in quiz on behavioral attributes.

	All Participants		Consistent Participants	
	OLS	Ordered Logit	OLS	Ordered Logit
	(1)	(2)	(3)	(4)
consistency	-0.110**	-0.285*		
	(0.056)	(0.155)		
switching point			-0.011	-0.033
			(0.017)	(0.047)
**I**{switch at 10}			0.382***	1.082***
			(0.128)	(0.354)
curiosity	0.015	0.038	0.029	0.077
	(0.017)	(0.046)	(0.021)	(0.058)
trust	-0.027*	-0.078*	-0.041**	-0.117**
	(0.016)	(0.044)	(0.019)	(0.053)
age	0.017***	0.047***	0.017***	0.046***
	(0.002)	(0.006)	(0.003)	(0.008)
female	0.198***	0.555***	0.196***	0.547***
	(0.094)	(0.147)	(0.063)	(0.174)
*R*^2^	0.119		0.089	
Pseudo *R*^2^		0.059		0.073
# obs	764	764	506	506

Notes: In the ordered logit models (columns 2 and 4), the dependent variable is ordered according to the number of false positives, with zero being the lowest and two being the highest. *, ** and *** denote significance at the 10%, 5%, and 1% level, respectively.

**Table 5:** Regression of number of false negatives in quiz on behavioral attributes.

	All Participants		Consistent Participants	
	OLS	Ordered Logit	OLS	Ordered Logit
	(1)	(2)	(3)	(4)
consistency	-0.114	-0.237		
	(0.084)	(0.150)		
switching point			-0.022	-0.047
			(0.026)	(0.047)
**I**{switch at 10}			0.217	0.369
			(0.190)	(0.348)
curiosity	0.015	0.027	0.042	0.078
	(0.025)	(0.046)	(0.031)	(0.057)
trust	0.008	0.010	0.003	-0.006
	(0.024)	(0.042)	(0.029)	(0.052)
age	-0.005	-0.007	-0.007	-0.011
	(0.003)	(0.006)	(0.004)	(0.008)
female	0.322***	0.631***	0.337***	0.723***
	(0.078)	(0.142)	(0.094)	(0.171)
*R*^2^	0.032		0.025	
Pseudo *R*^2^		0.015		0.018
# obs	764	764	506	506

Notes: In the ordered logit models (columns 2 and 4), the dependent variable is ordered according to the number of false negatives, with zero being the lowest and five being the highest. *** denotes significance at the 1% level, respectively.

By Result 1, we reject the null hypothesis in favor of Hypothesis 1 for consistent subjects. Our model is based on rational individuals who maximizes expected utility, therefore it does not make predictions about inconsistent subjects.

In short, our results for risk preference show that the ability to correctly identify a phishing attempt may relate to risk attitude in whether one is consistent, and if so, in whether one is willing to take any risk at all. Although option B provides a much higher expected payoff than option A in the latter lotteries, individuals who are intolerant of risk wait until the tenth and final lottery to switch, consistent with the finding that these individuals are more likely to make false positive errors. This result is consistent with [22], who find that risk attitude is predictive of one’s decision under the veil of ignorance.

An alternative interpretation of the correlation between consistency and quiz score is that they both come from the same unobservables, such as attention to the quiz and the games. Furthermore, one might be worried that switching in the last row in the lottery game reflects inattention or the use of heuristic rather than intolerance to risk. If this were the case, we would expect that extreme choices in the lottery game carry over to the other games. Table 6 provides the correlation between switching in the last row in the lottery game and extreme choices in other games. We first observe that intolerance of risk in the lottery game is significantly correlated with extreme choices in the gamble game in an economically meaningful way, i.e., positive correlation with the minimum, but negative correlation with the maximum, implying that both games measure the same underlying attributes they were designed to measure. However, we do not observe strong correlation between intolerance of risk with extreme choices in either the curiosity or the trust game. This analysis suggests that extreme choices in the lottery game does not extend to the measures of curiosity or trust.

**Table 6:** Correlation between intolerance of risk in the lottery game and extreme choices in other games.

	Gamble		Curiosity		Trust	
	min	max	min	max	min	max
Spearman’s *ρ*	0.224	-0.085	0.011	-0.020	0.015	-0.064
*p*-value	<0.001	0.019	0.754	0.588	0.676	0.092

Notes. In the gamble game, ‘min’ denotes choosing lottery 1 and ‘max’ denotes choosing lottery 9. In the curiosity game, ‘min’ denotes choosing 0 and ‘max’ denotes choosing all that one earns from the lottery game. In the trust game, ‘min’ denotes investing 0 and ‘max’ denotes investing 5.

### Curiosity

**Fig 3:**
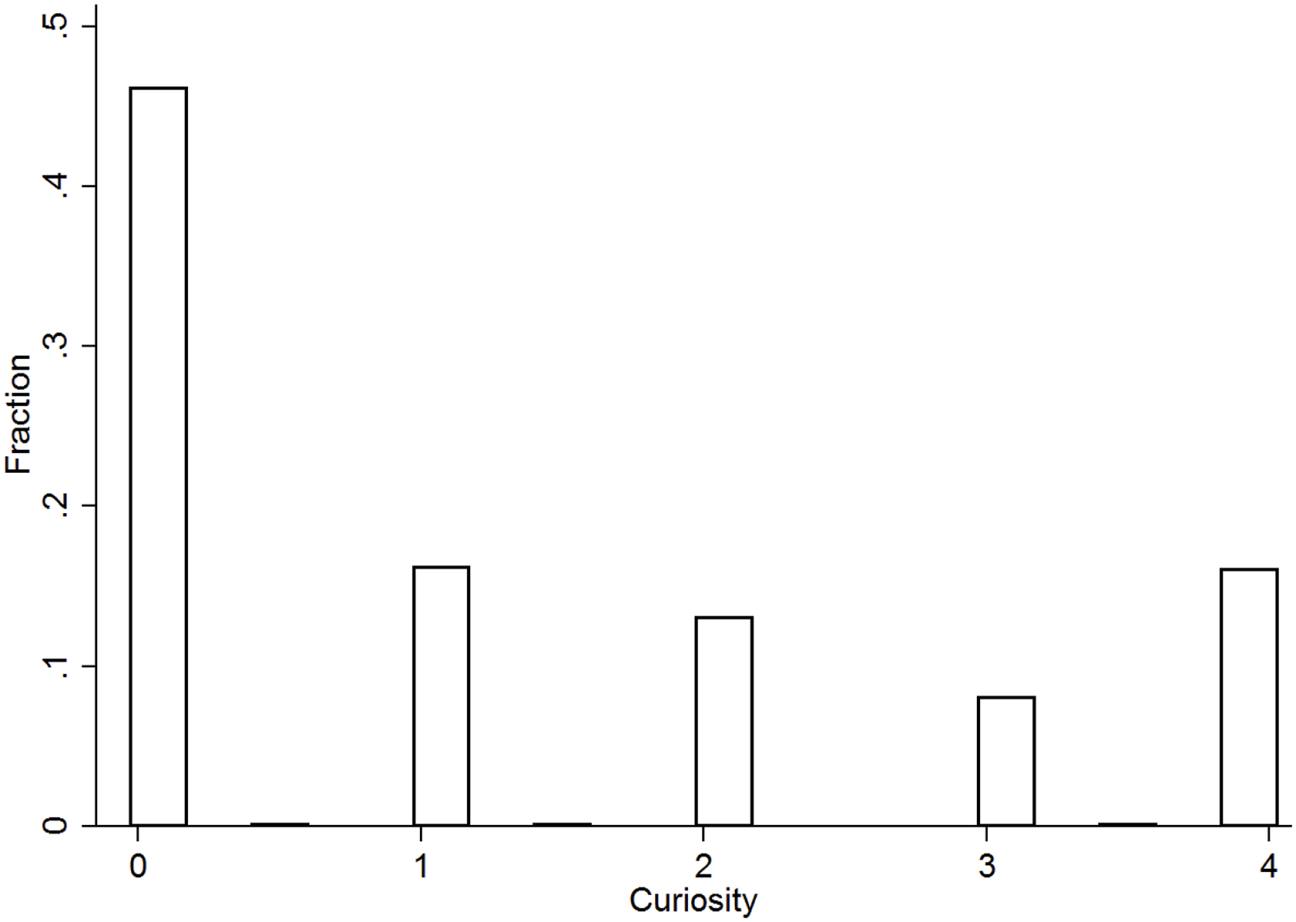
Distribution of willingness-to-pay for non-instrumental information (curiosity).

In our study, we measure curiosity by the willingness of a participant to pay for non-instrumental information, specifically the number of the lottery that was used to calculate her payoff. In Fig 3, we depict the distribution of subjects’ willingness to pay in our experiment. Our results show that 55% of our subjects are willing to pay a positive amount to learn which lottery was chosen to determine their payoff. This finding is consistent with those from previous literature related to the endogenous acquisition of information in the context of school choice [15], jury voting [23], and private value auctions [24], which show that subjects have a tendency to overinvest in information acquisition.

**Result 2** (Curiosity). Individuals who exhibit a greater level of curiosity perform worse in the security quiz.

**Support**. In Table 3, We report four regressions with curiosity as one of the independent variables. The results indicate that the number of correct answers on the quiz is negatively correlated to a participant’s level of curiosity (*p* < 0.05).

By Result 2, we reject the null in favor of Hypothesis 2.

### Trust

In our study, we measure how trusting our subjects are by the portion of a $5 endowment they are willing to transfer to another participant who then has the option to transfer money back to the first participant. Our results show that the subjects transfer an average of $2.85 (57% of their initial endowment) with a median of $3. Fewer than 10% of our subjects choose not to transfer any money. Compared with previous experiments of trust game where subjects invest 51% of their endowment on average (see the meta-analysis by [5]), our results indicate that the B&F staff members in our sample appear to be more trusting than those in the previous studies. In Table 3, we report the parameter estimates from the OLS regression with the amount of money invested as an independent variable.

**Result 3** (Trust). Individuals who are more trusting answer more questions correctly on the security quiz. Furthermore, when these individuals do misidentify an interface, they are more likely to regard a legitimate interface as a phishing attempt.

**Support**. The result in the first column of Table 3 shows that a participant’s quiz score is positively correlated with the amount of money she chooses to transfer. Regarding the regression with the false positives, we find that the parameter estimates are significantly less than zero (*p* < 0.05), indicating that those who transfer more money to a responder are less likely to misidentify legitimate interfaces as phishing attempts. Finally, we see that the regression using the number of false negatives generates a parameter estimate not significantly different from zero, suggesting that the level of trust does not relate to the tendency to commit false negatives.

By Result 3, we reject the null hypothesis in favor of Hypothesis 3(a), but fail to do so in favor of Hypothesis 3(b). Result 3 shows that trust has different predictive power on false positives and false negatives. Intuitively, a trusting individual puts herself in a potentially vulnerable position as the trustee might not return the amount invested. We might anticipate that trust has an effect on both false positives and false negatives. However, we only find the former, i.e., a more trusting individual is less likely to regard a legitimate website as a phishing attempt. In comparison, trust does not appear to be correlated with the tendency to commit false negative errors. That is, individuals who are less trusting are not less vulnerable to phishing attempts compared to their more trusting counterparts.

We further calculate for each subject the expected payoff given their choices as an investor in the trust game and find that individuals who are expected to earn a higher payoff performed better in the trust game. To the extent that trust is perceived as an investment decision with uncertainty [25], this result indicates that the subjects whose behavior is more consistent with expected payoff maximization tend to get a higher score in the security quiz. Furthermore, the analyses provide more insights regarding how trust relates to the number of false positives and the number of false negatives in the quiz. Those who have more trust in others are less likely to mark a legitimate website as phishing. That is, individuals who are not willing to give their own endowment for potential returns with uncertainty are more uncertain about legitimate websites and are hence more susceptible to false positive errors. Our results show that both intolerance to risk and trust serve as important predictors of vulnerability to phishing. This is reminiscent of [26], who show that although individuals face uncertainty in the trust game, trust serves as a different behavioral attribute from risk attitude in the decision-making process.

### Demographics

Finally, Table 3 provides the analysis of the relationship between the performance on the quiz and demographics.

**Result 4** (Age and gender). Older participants achieve a lower score on the security quiz. Additionally, female participants achieve a lower score than their male counterparts.

**Support**. The results in the last two rows of Table 3 show that the number of correct answers in the quiz is negatively correlated with participant age (-0.012, *p* < 0.05). This result supports previous findings that younger subjects are more likely to correctly identify phishing attempts [2, 27]. In addition, older participants are more likely to regard legitimate emails as phishing (0.019, *p* < 0.01). Regarding gender, the last row suggests that females score lower than males (-0.520, *p* < 0.01), with 36% attributed to false positives and 64% attributed to false negatives.

### Out-of-sample prediction of quiz performance

Our results so far establish that the behavioral attributes elicited from economic games correlate with the performance in the quiz. We now explore whether these behavioral attributes can provide out-of-sample prediction so that they can be used to diagnose individual susceptibility to phishing.

We follow the standard machine learning practice by randomly splitting the dataset into the training sample and the test sample in the ratio of 5:1. By construction, the training sample and the test sample follows the same distribution. The training sample is then used to estimate the prediction models and the test sample is used for evaluation. As the outcome variable is non-binary, we use the root mean square error as the main evaluation metric. The baseline prediction model is random guessing—the prediction value is randomly selected from the empirical distribution of the score in the test set.

To predict the score in the security quiz, we employ the model of random forest [28]. Random forest is based on regression trees and exploits the idea of random feature selection to avoid overfitting. We train our model using the 5-fold cross validation strategy on the training set. Table 7 presents the root mean square prediction error on both the training set and the test set. Compared to the baseline model, random forest gives lower prediction error both on the training set and the test set. Specifically, it reduces the root mean square from 1.841 to 1.248 (32%) for the in-sample prediction and 1.635 to 1.136 (31%) for the out-of-sample prediction.

**Table 7:** Root mean square prediction error.

	training set	test set
Baseline	1.841	1.635
Random Forest	1.248	1.136

**Fig 4:**
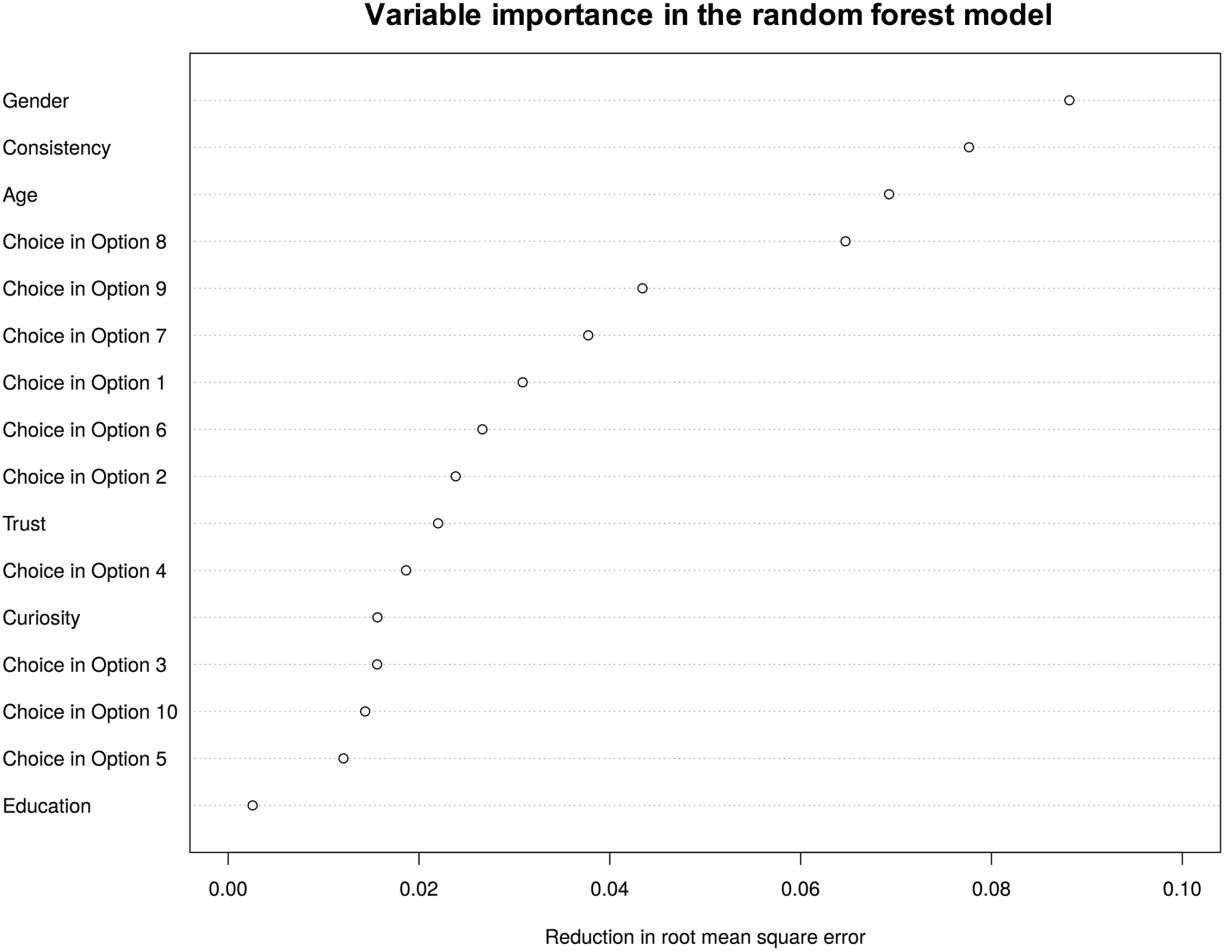
Variable importance in random forest.

Another advantage of the random forest model is that it provides a measure of how important a predictor is in predicting the outcome variable. The importance of a predictor is measured by the reduction in mean square prediction error when that predictor is used for tree growing. Fig 4 plots the variable important in the random forest model. It shows that gender, age, and consistency in the lottery choice are among the most important factors in out-of-sample predictions. It is notable that several of the features from economic games outperform education in predicting participants’ performance in the information security quiz.

## Conclusion

This paper explores how phishing susceptibility relates to measurable behavioral attributes, such as risk preference, curiosity, and trust. Using a lab-in-the-field experiment with staff members in the business and finance department of a large public university, we find that individuals who are intolerant of risk are more likely to regard legitimate interfaces as phishing. By contrast, we find that participants who are more trusting and less curious perform better on a security quiz. Our findings also reinforce those in previous research that older participants provide more incorrect answers as do females when compared to males. Finally, we provide a greater understanding of how behavioral traits relate to the ability to identify phishing attempts by decomposing the incorrect responses into false negatives and false positives. Our findings suggest the understanding of users’ ability to identify phishing attempts and suggest that the lottery choice game can be incorporated into design of phishing education programs and personalized security mitigation as a stable diagnostic tool. Future research can be focused on the tailored design of phishing awareness educational technologies based on these behavioral traits to reduce both false positives and false negatives. Because the behavioral traits elicited from the two games are shown to be stable in the literature, individual choices in the lottery game can also be used to identify potential victims to evolving phishing techniques and tailor the design of the anti-phishing education.

## Supporting information

S1 File (PDF)

S1 Table (PDF)

S2 Table (PDF)

S1 Fig (PDF)
