# Supplementary material for: Real or bogus: Predicting susceptibility to phishing with economic experiments
Source: PLoS One. 2018 Jun 27;13(6):e0198213. doi: 10.1371/journal.pone.0198213 (PMC6021067; doi:10.1371/journal.pone.0198213)
Supplement: S1 File — (PDF) [file pone.0198213.s001.pdf]

S1 File. The Security Quiz

1. You receive an email urging you to click the link below to renew your X Library privileges:

   `www.xxx.edu/renew-library-privileges`

   Is this a legitimate website?

2. Suppose you have a bank account with Wells Fargo and receive an email asking you to click the link below:

   `https://www.wellsfargo.com`

   Is this a legitimate link?

3. You receive an email that claims your library access will expire soon. It links to the site depicted. Is it safe to enter your username and password?

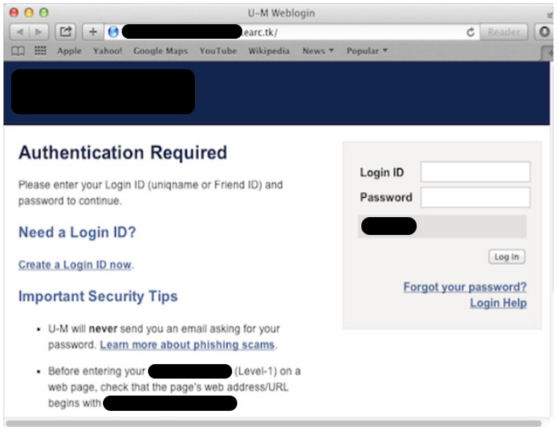

4. You have an account with Amazon and receive an email asking you to click the link below:

   `http://www.amazon.com.varzeas.us`

   Is this a legitimate link?

5. You have an account with Citibank and receive an email asking you to click the link below:

   `http://www.citibanking.net`

   Is this a legitimate link?

6. Suppose Tom received an email from a friend that says a document has been shared with him on Dropbox. The email is shown below. Do you think Tom should click the ”view folder” button?

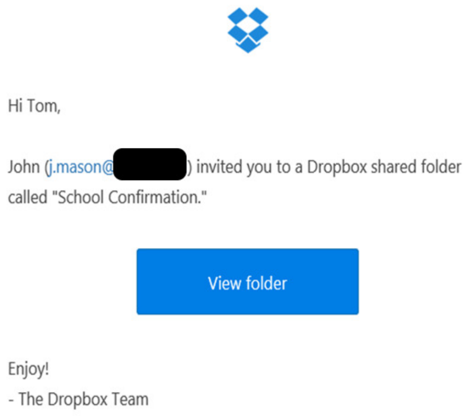

7. You have an account with Amazon and receive an email asking you to click the link below:

   `http://www.amazon.co.uk`

   Is this a legitimate link?
