# Supplementary material for: Real or bogus: Predicting susceptibility to phishing with economic experiments
Source: PLoS One. 2018 Jun 27;13(6):e0198213. doi: 10.1371/journal.pone.0198213 (PMC6021067; doi:10.1371/journal.pone.0198213)
Supplement: S1 Table — (PDF) [file pone.0198213.s002.pdf]

S1 Table. Odds Ratio in the Ordered Logit Models

	All Participants			Consistent Participants		
	Average Score	False Positives	False Negatives	Average Score	False Positives	False Negatives
consistency	0.224**	0.752*	0.789			
	(0.100)	(0.117)	(0.118)			
switching point				1.056	0.968	0.954
				(0.048)	(0.046)	(0.044)
**I**{switch at 10}				0.454**	2.951***	1.446
				(0.149)	(1.045)	(0.503)
curiosity	-0.030	1.039	1.027	0.887**	1.081	1.081
	(0.030)	(0.048)	(0.047)	(0.050)	(0.062)	(0.062)
trust	0.019	0.925*	1.010	1.051	0.890**	0.995
	(0.028)	(0.041)	(0.043)	(0.052)	(0.047)	(0.051)
age	-0.012***	1.048***	0.993	0.982**	1.047***	0.989
	(0.004)	(0.007)	(0.006)	(0.007)	(0.008)	(0.007)
female	-0.520***	1.742***	1.880***	0.415***	1.729***	2.061***
	(0.094)	(0.255)	(0.267)	(0.069)	(0.302)	(0.352)
R^2	0.080	0.059	0.015	0.033	0.073	0.018
# obs	764	764	764	506	506	506

Notes: Odds ratios are reported. Values larger (smaller) than one indicate positive (negative) effect. *, **and ***denote significance at the 10%, 5%, and 1% level, respectively.
