# Supplementary material for: Real or bogus: Predicting susceptibility to phishing with economic experiments
Source: PLoS One. 2018 Jun 27;13(6):e0198213. doi: 10.1371/journal.pone.0198213 (PMC6021067; doi:10.1371/journal.pone.0198213)
Supplement: S2 Table — (PDF) [file pone.0198213.s003.pdf]

S2 Table. Decisions in the trust game from the pilot study

Subject No.	Investment	Return 0	Return 1	Return 2	Return 3	Return 4	Return 5
1	5	0	0	0	1	1	2
2	5	0	2	4	7	9	10
3	3	0	1	2	4	5	7
4	0	0	0	0	0	0	0
5	2	0	0	0	1	0	5
6	4	0	2	3	6	8	8
7	5	5	3	2	3	7	10
8	3	0	2	3	4	6	8
9	1	0	0	0	0	0	0
10	2	2	2	1	3	3	5
11	0	0	2	4	6	8	10
12	5	0	1	3	5	7	10
13	2	0	1	2	3	5	5
14	2	2	3	5	7	8	10
15	1	0	0	0	0	0	0
16	2	0	2	4	6	7	10
17	1	0	0	0	3	5	7
18	3	0	2	4	5	7	8
19	5	3	1	1	2	4	6
20	2	0	1	2	3	4	5
21	1	5	7	0	1	3	5
22	1	0	1	3	4	6	8
23	1	1	2	4	6	8	10
24	1	0	1	1	1	1	1
25	3	1	1	1	2	2	2
26	2	1	1	3	4	5	9
27	2	0	1	2	4	6	10
28	0	0	1	4	6	8	10
29	0	1	3	4	3	4	5
30	2	0	1	4	5	7	10
31	0	0	1	2	3	4	5
32	0	0	0	0	0	0	0
33	1	1	2	3	2	2	2
34	4	2	2	2	4	5	4
35	2	2	3	4	4	5	7
36	4	3	5	7	9	10	11
37	5	5	8	11	7	8	10
38	5	0	0	0	0	0	6
39	1	0	2	0	0	0	0
40	1	0	1	2	3	4	5
41	3	1	2	4	6	9	11
