# Supplementary material for: Real or bogus: Predicting susceptibility to phishing with economic experiments
Source: PLoS One. 2018 Jun 27;13(6):e0198213. doi: 10.1371/journal.pone.0198213 (PMC6021067; doi:10.1371/journal.pone.0198213)
Supplement: S1 Fig — (PDF) [file pone.0198213.s004.pdf]

S1 Fig. Interfaces for the Economics Games

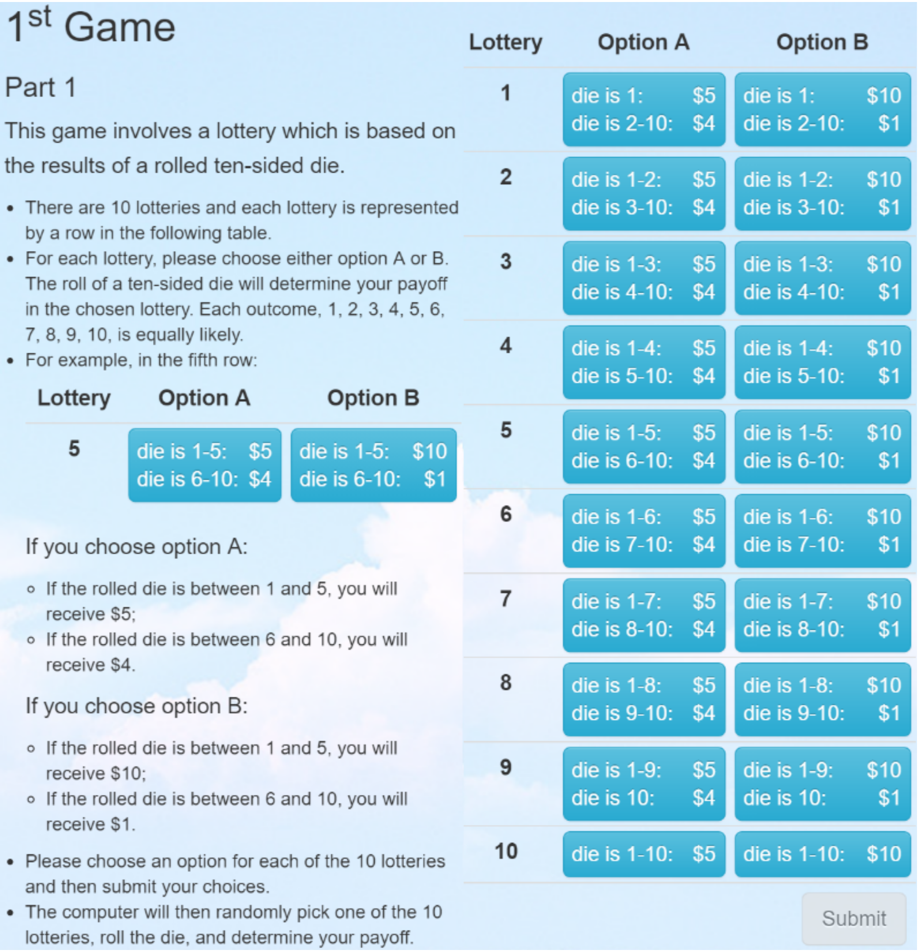

1st Game

Part 1

This game involves a lottery which is based on the results of a rolled ten-sided die.

- There are 10 lotteries and each lottery is represented by a row in the following table.
- For each lottery, please choose either option A or B. The roll of a ten-sided die will determine your payoff in the chosen lottery. Each outcome, 1, 2, 3, 4, 5, 6, 7, 8, 9, 10, is equally likely.
- For example, in the fifth row:

Lottery	Option A	Option B
5	die is 1-5: $5<br>die is 6-10: $4	die is 1-5: $10<br>die is 6-10: $1

If you choose option A:

- If the rolled die is between 1 and 5, you will receive $5;
- If the rolled die is between 6 and 10, you will receive $4.

If you choose option B:

- If the rolled die is between 1 and 5, you will receive $10;
- If the rolled die is between 6 and 10, you will receive $1.

- Please choose an option for each of the 10 lotteries and then submit your choices.
- The computer will then randomly pick one of the 10 lotteries, roll the die, and determine your payoff.

Lottery	Option A	Option B
1	die is 1: $5<br>die is 2-10: $4	die is 1: $10<br>die is 2-10: $1
2	die is 1-2: $5<br>die is 3-10: $4	die is 1-2: $10<br>die is 3-10: $1
3	die is 1-3: $5<br>die is 4-10: $4	die is 1-3: $10<br>die is 4-10: $1
4	die is 1-4: $5<br>die is 5-10: $4	die is 1-4: $10<br>die is 5-10: $1
5	die is 1-5: $5<br>die is 6-10: $4	die is 1-5: $10<br>die is 6-10: $1
6	die is 1-6: $5<br>die is 7-10: $4	die is 1-6: $10<br>die is 7-10: $1
7	die is 1-7: $5<br>die is 8-10: $4	die is 1-7: $10<br>die is 8-10: $1
8	die is 1-8: $5<br>die is 9-10: $4	die is 1-8: $10<br>die is 9-10: $1
9	die is 1-9: $5<br>die is 10: $4	die is 1-9: $10<br>die is 10: $1
10	die is 1-10: $5	die is 1-10: $10

Submit

Figure 1: Lottery Choice Game

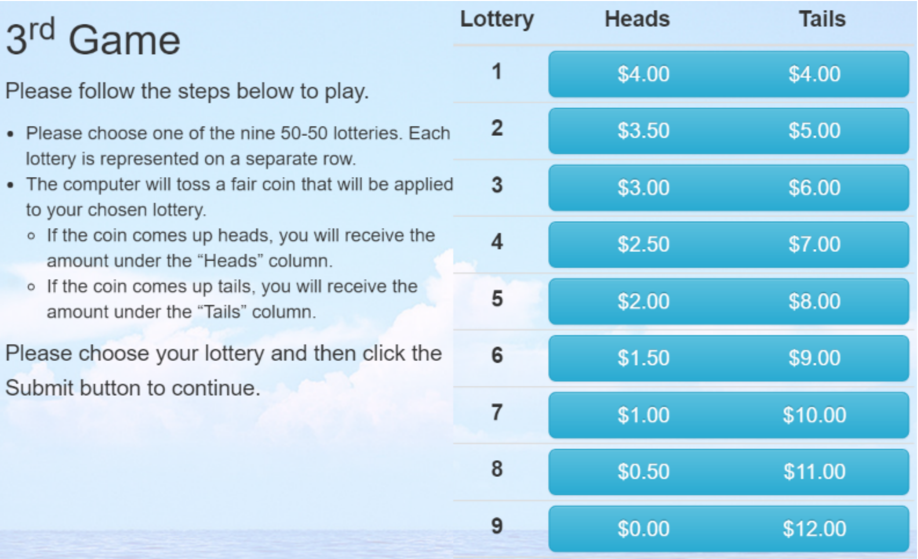

Figure 2: Gamble Game

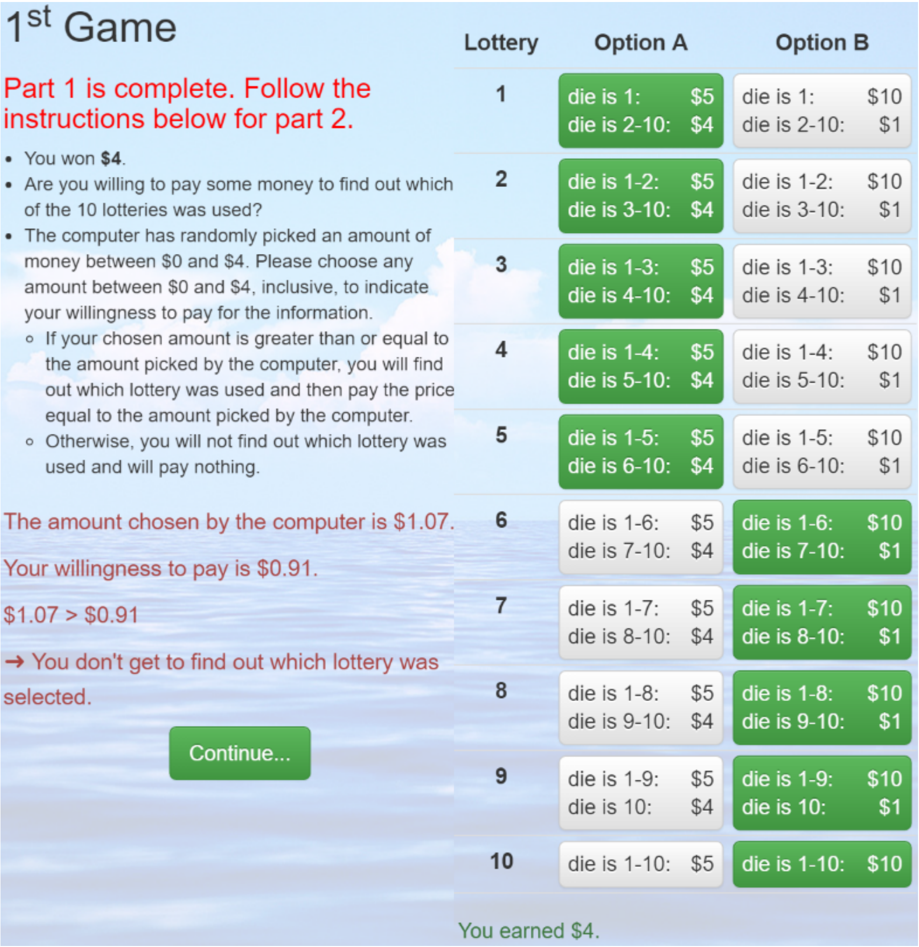

Figure 3: Curiosity Measure

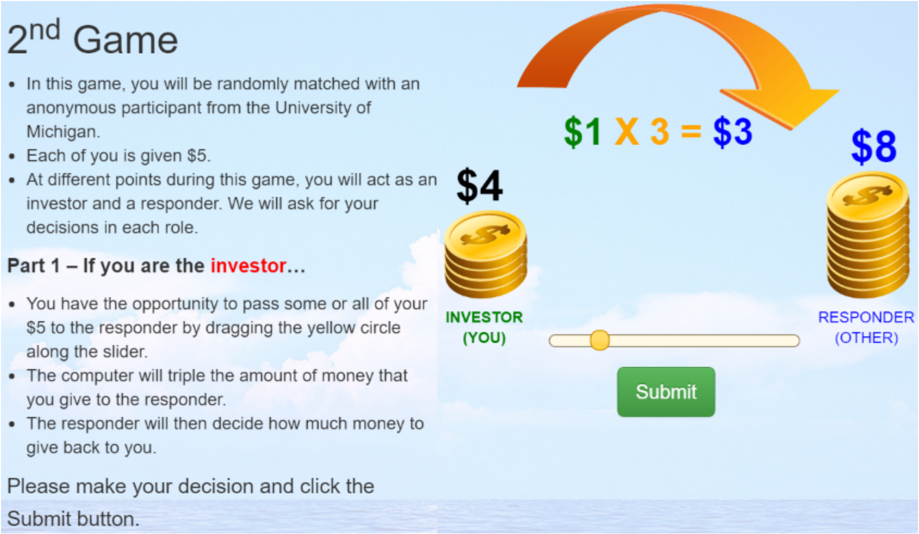

Figure 4: Trust Game (Investor)

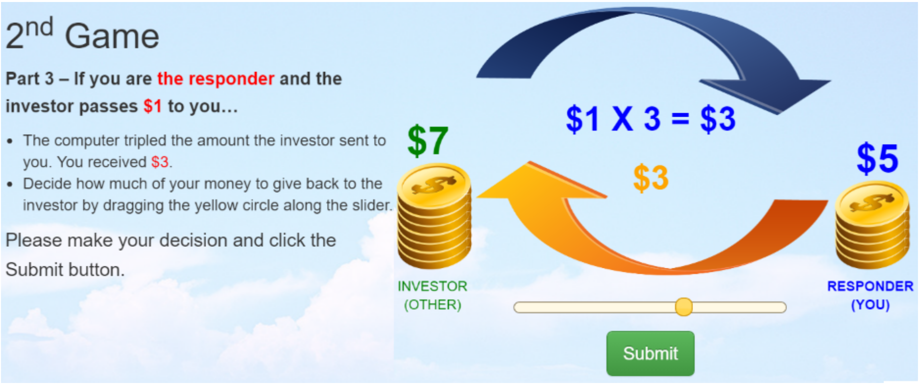

Figure 5: Trust Game (Responder)
